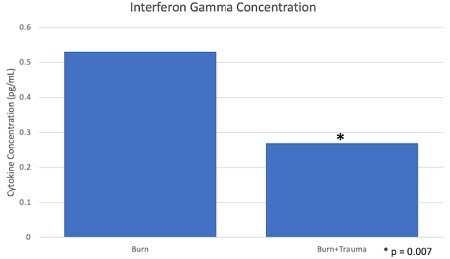# 808 The Inflammatory Response of Patients with Major Burns and Traumatic Injuries

**DOI:** 10.1093/jbcr/irae036.348

**Published:** 2024-04-17

**Authors:** Jared M Robinson, Sophia Trinh, Jenna Dennis, Cameron Fontenot, Jeffery Hobden, Jonathan E Schoen, Herb A Phelan, Jeffrey E Carter, Alison A Smith

**Affiliations:** Louisiana State University Health Sciences Center, New Orleans, New Orleans, Louisiana; Louisiana State University, New Orleans, LA; University Medical Center (LSU Health), New Orleans, LA; Louisiana State University Health Sciences Center- New Orleans, New Orleans, LA; Louisiana State University, New Orleans, LA; Louisiana State University Health Sciences Center, New Orleans, New Orleans, Louisiana; Louisiana State University, New Orleans, LA; University Medical Center (LSU Health), New Orleans, LA; Louisiana State University Health Sciences Center- New Orleans, New Orleans, LA; Louisiana State University, New Orleans, LA; Louisiana State University Health Sciences Center, New Orleans, New Orleans, Louisiana; Louisiana State University, New Orleans, LA; University Medical Center (LSU Health), New Orleans, LA; Louisiana State University Health Sciences Center- New Orleans, New Orleans, LA; Louisiana State University, New Orleans, LA; Louisiana State University Health Sciences Center, New Orleans, New Orleans, Louisiana; Louisiana State University, New Orleans, LA; University Medical Center (LSU Health), New Orleans, LA; Louisiana State University Health Sciences Center- New Orleans, New Orleans, LA; Louisiana State University, New Orleans, LA; Louisiana State University Health Sciences Center, New Orleans, New Orleans, Louisiana; Louisiana State University, New Orleans, LA; University Medical Center (LSU Health), New Orleans, LA; Louisiana State University Health Sciences Center- New Orleans, New Orleans, LA; Louisiana State University, New Orleans, LA; Louisiana State University Health Sciences Center, New Orleans, New Orleans, Louisiana; Louisiana State University, New Orleans, LA; University Medical Center (LSU Health), New Orleans, LA; Louisiana State University Health Sciences Center- New Orleans, New Orleans, LA; Louisiana State University, New Orleans, LA; Louisiana State University Health Sciences Center, New Orleans, New Orleans, Louisiana; Louisiana State University, New Orleans, LA; University Medical Center (LSU Health), New Orleans, LA; Louisiana State University Health Sciences Center- New Orleans, New Orleans, LA; Louisiana State University, New Orleans, LA; Louisiana State University Health Sciences Center, New Orleans, New Orleans, Louisiana; Louisiana State University, New Orleans, LA; University Medical Center (LSU Health), New Orleans, LA; Louisiana State University Health Sciences Center- New Orleans, New Orleans, LA; Louisiana State University, New Orleans, LA; Louisiana State University Health Sciences Center, New Orleans, New Orleans, Louisiana; Louisiana State University, New Orleans, LA; University Medical Center (LSU Health), New Orleans, LA; Louisiana State University Health Sciences Center- New Orleans, New Orleans, LA; Louisiana State University, New Orleans, LA

## Abstract

**Introduction:**

Major burns resulting from high impact mechanisms such as explosions are commonly associated with other traumatic injuries. It is known that the inflammatory response to burns and traumatic injuries are altered when observed in isolation. However, the inflammatory response of major burns with concomitant traumatic injuries is not well studied. Adipose-derived stem cells (ADSCs) are a multipotent stem cell and play an important role in the immunomodulatory response and wound healing. One mechanism they exert these effects is by secreting certain cytokines. Studies have shown alterations in the cytokine profile of major burn patients. However, no studies have been performed evaluating the cytokines released by burn wounds in patients with concomitant traumatic injuries. We hypothesized that there is an alteration in the paracrine factors secreted by ADSCs in patients with major burn wounds and concomitant traumatic injuries.

**Methods:**

Adipose tissue was collected from patients with severe burn injuries (>20% total body surface area, TBSA) at their index operation and ADSCs were extracted. Fluorescence activated single cell sorting () confirmed the presence of ADSCs. ADSCs were grown under standard tissue culture techniques. The supernatant was extracted. Cytokine analyses were performed with multiplex assays. Each subject’s chart was analyzed for a concomitant traumatic injury. A student’s t test was used to analyze the groups.

**Results:**

A total of 21 patients were enrolled in the study, 2 had concomitant traumatic injuries. One patient sustained a subarachnoid hemorrhage, pulmonary contusions, and lower extremity fractures and the other patient sustained upper extremity fractures. There was a significant difference in the mean TBSA burned between the two groups (injuries 50%, no injuries 31%; p< 0.05). There was no significant difference in the gender, age, body mass index, or race (p>0.05). There was a significantly lower concentration of interferon gamma in patients with concomitant traumatic injuries.

**Conclusions:**

This study demonstrated a significantly lower concentration of interferon gamma in burn patients with concomitant traumatic injuries. Interferon gamma is an important immunomodulator. It’s functions include macrophage activation, mediating antiviral and antibacterial immunity, and plays a role in the activation of the innate immune system. Further research is warranted to investigate the role of interferon gamma in traumatic burn patients.

**Applicability of Research to Practice:**

The results of this study can be applied to practice because it demonstrates a significant difference in the concentration of interferon gamma in patients with major burns and concomitant traumatic injuries. According to some studies, interferon gamma has been shown to delay wound healing by inhibiting collagen synthesis. This knowledge could allow clinicians to improve management of burn injuries in this patient population.